# Evolution of hepatitis A virus seroprevalence among HIV-positive adults in Taiwan

**DOI:** 10.1371/journal.pone.0186338

**Published:** 2017-10-16

**Authors:** Yu-Lin Lee, Kuan-Yin Lin, Chien-Yu Cheng, Chia-Wen Li, Chia-Jui Yang, Mao-Song Tsai, Hung-Jen Tang, Te-Yu Lin, Ning-Chi Wang, Yi-Chien Lee, Shih-Ping Lin, Yu-Shan Huang, Hsin-Yun Sun, Jun-Yu Zhang, Wen-Chien Ko, Shu-Hsing Cheng, Yuan-Ti Lee, Chun-Eng Liu, Chien-Ching Hung, on behalf of the Taiwan HIV Study Group

**Affiliations:** 1 Department of Internal Medicine, Changhua Christian Hospital, Changhua, Taiwan; 2 Department of Medicine, National Taiwan University Hospital Jin-Shan Branch, New Taipei City, Taiwan; 3 Department of Infectious Diseases, Taoyuan General Hospital, Ministry of Health and Welfare, Taoyuan, Taiwan; 4 School of Public Health, National Yang-Ming University, Taipei, Taiwan; 5 Department of Internal Medicine, National Cheng Kung University Hospital, Tainan, Taiwan; 6 Department of Medicine, National Cheng Kung University Medical College, Tainan, Taiwan; 7 Department of Internal Medicine, Far Eastern Memorial Hospital, New Taipei City, Taiwan; 8 School of Medicine, National Yang-Ming University, Taipei, Taiwan; 9 Department of Internal Medicine, Chi Mei Medical Center, Tainan, Taiwan; 10 Department of Health and Nutrition, Chia Nan University of Pharmacy and Sciences, Tainan, Taiwan; 11 Department of Internal Medicine, Tri-Service General Hospital and National Defense Medical Center, Taipei, Taiwan; 12 Department of Internal Medicine, Ditmanson Medical Foundation Chia-Yi Christian Hospital, Chia-Yi, Taiwan; 13 Department of Internal Medicine, Taichung Veterans General Hospital, Taichung, Taiwan; 14 Department of Internal Medicine, National Taiwan University Hospital Hsin-Chu Branch, Hsin-Chu, Taiwan; 15 Department of Internal Medicine, National Taiwan University Hospital, and National Taiwan University College of Medicine, Taipei, Taiwan; 16 Center of Infection Control, National Taiwan University Hospital, Taipei, Taiwan; 17 School of Public Health, Taipei Medical University, Taipei, Taiwan; 18 School of Medicine, Chung Shan Medical University, Taichung, Taiwan; 19 Department of Internal Medicine, Chung Shan Medical University Hospital, Taichung, Taiwan; 20 Department of Parasitology, National Taiwan University College of Medicine, Taipei, Taiwan; Centers for Disease Control and Prevention, UNITED STATES

## Abstract

**Objectives:**

The study aimed to describe the seroprevalence of hepatitis A virus (HAV) in HIV-positive adult patients in Taiwan between 2012 and 2016 and to examine the evolution of HAV seroprevalence between 2004–2007 and 2012–2016.

**Methods:**

Clinical information and data of anti-HAV antibody results were collected from 2,860 antiretroviral-naïve HIV-positive Taiwanese aged 18 years or older who initiated combination antiretroviral therapy at 11 hospitals around Taiwan between 2012 and 2016 (2012–2016 cohort). A multivariate logistic regression model was applied to identify independent variables associated with HAV seropositivity. Comparisons of HAV seroprevalences and associated clinical characteristics were made between this 2012–2016 cohort and a previous cohort of 1580 HIV-positive patients in 2004–2007 (2004–2007 cohort).

**Results:**

Of the 2,860 HIV-positive patients between 2012 and 2016, the overall HAV seropositivity rate was 21.2% (605/2860), which was independently associated with an older age (adjusted odds ratio [AOR], per 1-year increase, 1.13; 95% confidence interval [95% CI], 1.11–1.15) and co-infection with hepatitis B virus (AOR 1.44; 95% CI, 1.08–1.93). Residence in southern Taiwan (AOR 0.49; 95% CI, 0.34–0.72) was inversely associated with HAV seropositivity. The overall HAV seroprevalence in the 2012–2016 cohort was significantly lower than that in the 2004–2007 cohort (21.2% vs 60.9%, p<0.01). The decreases of HAV seropositivity rate were observed in nearly every age-matched group, which suggested the cohort effect on HAV seroepidemiology. However, among individuals aged 25 years or younger, the HAV seropositivity rate increased from 3.8% (2/52) in the 2004–2007 cohort to 8.5% (50/587) in the 2012–2016 cohort, with 95.4% (560/587) being MSM in this age group of the latter cohort.

**Conclusions:**

HAV seroprevalence has decreased with time among HIV-positive adults in Taiwan. The cohort effect has increased the number of young HIV-positive patients that are susceptible to HAV infection in a country without nationwide childhood vaccination program against HAV.

## Introduction

Hepatitis A virus (HAV) is transmitted through the fecal-oral route either by direct contact with an infectious person or by ingestion of contaminated food or water [1]. According to the World Health Organization (WHO) estimation, HAV infection caused 3.7 million illnesses and 28,000 deaths in 2010 with differences observed in regions of different endemicities around the world [2]. In the developing countries in Asia, Africa, Central and South Americas, and Oceania, most HAV infections occur in childhood and the seroprevalence before teenage ranges from 63% to 94% [3, 4]. In contrast, the overall HAV seroprevalence is less than 15% among the adolescents in the North America, Europe, and Australia [5].

The correlation between the HIV and HAV infection varies according to the local HIV and HAV epidemiology [6]. In the countries of high HAV endemicity, no significant difference of HAV seroprevalence was observed between HIV-positive and HIV-negative individuals [7]. In contrast, HIV-positive patients usually have a higher HAV seroprevalence than their HIV-negative counterparts in the developed countries of low HAV endemicity [8, 9]. Certain sexual behaviors associated with risk groups for HIV transmission may also increase the risk for HAV transmission, including oral-anal sex [10] and percutaneous exposure to contaminated illicit drugs or injecting equipment [11]. Those risky behaviors may facilitate the emergence of acute hepatitis A outbreaks in countries of low HAV endemicity because of an increasing number of susceptible hosts. For example, injecting drug users (IDUs) in countries with better health and hygiene conditions usually have higher HAV seroprevalence than the general population [12–15], and acute HAV infection among IDUs may be associated with a higher fatality rate due to co-infections with hepatitis B virus (HBV) or hepatitis C virus (HCV) [11]. On the other hand, outbreaks of acute HAV infection among men who have sex with men (MSM) emerged in recent years across several European countries and the UK [16–18]. The outbreaks were linked to risky sexual contacts and increased international travel and susceptible hosts, especially in young adults with low adherence to recommended HAV vaccination [6, 17].

Taiwan used to be endemic for HAV infection; seroepidemiologic studies in the 1980s revealed that more than 90% of Taiwanese became HAV-seropositive by the age of 20 years and the rates increased with age [19–21]. With the improvement of sanitation and hygiene and implementation of vaccination program for children in counties of high endemicity for HAV infection, HAV seroprevalence has significantly declined and less than 10% of teenagers were positive for anti-HAV antibodies in 1998 [22]. Among the HIV-positive patients, a study by Sun et al between 2004 and 2007, when an outbreak of HIV infection was occurring among IDUs in Taiwan, revealed a higher HAV seroprevalence (60.9%) among HIV-positive patients than HIV-negative individuals seeking health check-ups (48.0%); and injection drug use and age were identified as the independent factors associated with HAV seropositivity [9].

However, the HIV epidemic has significantly changed in recent years in Taiwan. The proportion of IDUs among the newly diagnosed HIV-positive patients declined rapidly with the successful, sustained implementation of harm reduction program since 2004–2005 [23–25]; instead, the number of young HIV-positive MSM increased steadily and MSM has again become the major risk group for HIV transmission. The increasing number of susceptible host who have a low adherence to the recommendations of HAV vaccination has laid the fertile ground for an outbreak of acute hepatitis A to occur in Taiwan in June 2015 [26–28]. Furthermore, the outbreak strain in Taiwan appeared to be genetically linked to the strain that caused the recent outbreak of acute hepatitis A in Europe [17].

In this study, we aimed to examine the evolution of HAV seroepidemiology and identify the associated factors with HAV infection among HIV-positive Taiwanese patients in recent years to help inform the HAV vaccination policy.

## Patients and methods

### Study setting and population

This retrospective cohort study was conducted at 11 major designated hospitals for HIV care around Taiwan, where HIV care including combination antiretroviral therapy (cART) and monitoring of CD4 and plasma HIV RNA load (PVL) has been provided free-of-charge [29]. Between 1 June, 2012 and 31 May, 2016, all patients who were aged 18 years or greater and initiated cART were included in this study (2012–2016 cohort). A case record form was used to collect information on the demographic and clinical characteristics of the patients at baseline and during follow-up, which included birth date, sex, route of HIV transmission, serologies of viral hepatitis and syphilis, CD4, and PVL. Patients were divided into four risk groups according to the routes of HIV transmission including MSM, IDUs, heterosexual contact, and unknown status. The study was approved by the Research Ethics Committee of National Taiwan University Hospital [201003112R] and Far Eastern Memorial Hospital [105040-F], Medical Ethics and Institutional Review Board of Taoyuan General Hospital [TYGH103011], and Institutional Review Boards of Tri-Service General Hospital [1-105-05-057], National Taiwan University Hospital Hsin-Chu Branch [105-017-F], Taichung Veterans General Hospital [CF16114B], Chung Shan Medical University Hospital [CS14034], Changhua Christian Hospital [160408], Chia-Yi Christian Hospital [105034], National Cheng Kung University Hospital [B-BR-105-038], and Chi Mei Medical Center [10505–002]. The informed consent was waived.

HAV vaccination was not included in routine vaccination schedule for toddlers in Taiwan. Since 1995, HAV vaccination was only provided to children in 30 indigenous townships and 19 non-indigenous townships in order to control HAV infection in counties of high endemicities of HAV infection in Taiwan. However, the vaccination program covered only 2% of the total population in Taiwan. The study sites participating in this study did not include hospitals located in those townships that were covered by the HAV vaccination program. It was only until one year after the onset of the recent outbreak of acute hepatitis A that Taiwan Centers for Disease Control (CDC) started to implement free-of-charge 1-dose HAV vaccination program in October 2016 that aimed to target those testing negative for anti-HAV antibody who were aged 40 years or less, those who had close contacts with individuals receiving the diagnosis of acute hepatitis A, and those who had sexually transmitted infections.

A previous retrospective cohort study conducted at two designated hospitals for HIV care by Sun et al was compared to examine the evolution of HAV seroprevalence among HIV-positive patients in Taiwan [9]. The previous cohort study was performed between 2004 and 2007 (2004–2007 cohort), which included 1580 HIV-positive persons seeking HIV care and 2581 HIV-negative controls seeking health check-up at the National Taiwan University Hospital, Taipei (northern Taiwan) and National Taiwan University Hospital Yun-Lin Branch (central Taiwan). In this 2004–2007 cohort, the HIV-positive patients included 658 (41.6%) MSM, 304 (19.2%) heterosexuals, and 577 (36.5%) IDU.

### Laboratory investigations

Testing for anti-HIV antibodies, Western blot for confirmation of HIV infection, CD4 count and PVL and antibodies for HBV and HCV were performed by each participating hospital with the use of certified diagnostic kits [29]. Data on baseline CD4 count and PVL, and baseline anti-HAV antibody, anti-HCV antibody, hepatitis B surface antigen (HBsAg), anti-hepatitis B surface antibody (anti-HBs antibody), anti-hepatitis B core antibody (anti-HBc antibody), and the rapid plasma reagin (RPR) for syphilis were collected locally at each participating hospital, which were then pooled and analyzed at the National Taiwan University Hospital, Taipei. HBV infection was defined as patients to have positive result of either HBsAg or anti-HBc antibody or both.

### Statistical analysis

All statistical analyses were performed with the use of SPSS software version 20.0 (SPSS Inc., Chicago, IL, USA). Categorical variables were compared using the Chi-square or Fisher’s exact test, whereas non-categorical variables were compared using the Mann-Whitney U-test. The variables with p-value <0.2 in univariate analysis were allowed to enter into multiple logistic regression model. A multiple logistic regression model was built to identify independent variables associated with anti-HAV seropositivity. All tests were two tailed and a p-value of <0.05 was considered significant.

## Results

In the 2012–2016 cohort, a total of 2,860 HIV-positive patients were included for analysis, which included 2,229 (77.9%) MSM, 437 (15.3%) IDUs, 166 (5.8%) heterosexuals, and 28 (1.0%) others (Table 1). MSM (mean age, 30.5 ± 7.7 years) were significantly younger than heterosexuals (40.0 ± 12.5 years) and IDUs (41.8 ± 7.7 years) (both comparisons, p<0.001). With regard to specific age groups, 54.9% of MSM were aged less than 30 years, while only 24.1% of heterosexuals and 2.8% of IDUs were aged less than 30 years (both comparisons, p<0.001). The overall HAV seroprevalence was 21.2% and the rate was significantly lower among MSM (16.7%) than heterosexuals (37.3%) and IDU (37.1%) (both comparisons, p<0.001). In our study, most of the included patients lived in northern Taiwan (70.4%), and the composition of risk groups for HIV infection, including MSM, heterosexuals and IDUs, was different across the different regions in Taiwan. For example, less MSM (154/316, 48.7%) and more IDUs (123/316, 38.9%) were included in central Taiwan than northern Taiwan (MSM, 81.8% [1647/2013]; and IDUs, 13.7% [276/2013]) and southern Taiwan (MSM, 80.6% [428/531]; and IDUs 7.1% [38/531]) (both comparisons, p<0.001, Table 1).

**Table 1:** Demographic and clinical characteristics of HIV-positive patients with different routes of HIV transmission, 2012–2016.

	All HIV-positive persons	Men who have sex with men	Heterosexuals	Injecting drug users
Number, n =	2860	2229	166	437
Male sex, n (%)	2730 (95.5)	2229 (100)	107 (64.5)	368 (84.2)
Age, mean ± SD, years^a^	32.9 ± 9.3	30.5 ± 7.7	40.0 ± 12.5^c^	41.8 ± 7.7^c^
Age group, years^a^				
18–20, n (%)	78 (2.7)	75 (3.4)	3 (1.8)	0 (0.0)
21–25	509 (17.8)	485 (21.8)	16 (9.6)	2 (0.5)
26–30	696 (24.3)	661 (29.7)	21 (12.7)	10 (2.3)
31–35	606 (21.2)	497 (22.3)	29 (17.5)	75 (17.2)
36–40	382 (13.4)	229 (10.3)	24 (14.5)	124 (28.4)
41–45	255 (8.9)	153 (6.9)	12 (7.2)	88 (20.1)
46–50	173 (6.0)	84 (3.8)	24 (14.5)	64 (14.6)
51–55	86 (3.0)	25 (1.1)	14 (8.4)	46 (10.5)
56–60	55 (1.9)	15 (0.7)	11 (6.6)	26 (5.9)
>60	20 (0.7)	5 (0.2)	12 (7.2)	2 (0.5)
Residence in Taiwan^a^, n (%)				
Northern	2013 (70.4)	1647 (73.9)	75 (45.2)	276 (63.2)
Central	316 (11.0)	154 (6.9)	29 (17.5)	123 (28.1)
Southern	531 (18.6)	428 (19.2)	62 (37.3)	38 (8.7)
Cohort				
Diagnosed before 2015/5/31	2118 (74.1)	1642 (73.7)	128 (77.1)	327 (74.8)
Diagnosed after 2015/6/1	742 (25.9)	587 (26.3)	38 (22.9)	110 (25.2)
Median CD4,^a^ (range), cells/μl	272 (0–2217)	275 (0–2217)	216 (1–1085)^c^	287 (0–917)
Median plasma HIV RNA load,^a^ (range), log10, copies/ml	4.8 (1.3–7.6)	4.8 (1.43–7.6)	4.7 (1.6–6.6)	4.4 (1.3–6.7)^c^
Positive anti-HAV antibody, n (%)	605 (21.2)	372 (16.7)	62 (37.3)^c^	162 (37.1)^c^
Positive HBsAg, n/N^b^ (%)	309/2851 (10.8)	200/2221 (9.0)	24/165 (14.5)^c^	82/437 (18.8)^c^
Positive anti-HBs antibody, n/N^b^ (%)	1083/2051 (52.8)	927/1720 (53.9)	64/135 (47.4)	86/179 (48.0)
Positive anti-HBc antibody, n/N^b^ (%)	603/1937 (31.1)	430/1629 (26.4)	50/123 (40.7)^c^	119/170 (70.0)^c^
Positive anti-HCV antibody, n/N^b^ (%)	511/2849 (17.9)	78/2219 (3.5)	20/166 (12.0)^c^	412/436 (94.5)^c^

**Abbreviations:** anti-HAV, anti-hepatitis A virus; anti-HBs, anti-hepatitis B surface; anti-HBc, anti-hepatitis B core; anti-HCV, anti-hepatitis C virus; HBsAg, hepatitis B surface antigen

^a^ p<0.001 for comparisons among men who have sex with men (MSM), heterosexuals, and injecting drug users (IDUs); between MSM and heterosexuals; between MSM and IDUs; and between heterosexuals and IDUs.

^b^ n/N = number of patients with positive test results/number of patients with test results.

^c^ Compared with MSM, p<0.05.

For co-infections with other viral hepatitis, MSM had statistically significantly lower seropositive rates of HBsAg (9.0%) and anti-HBc antibody (26.4%) than heterosexuals and IDUs, and higher percentages of positive anti-HBs antibodies than non-MSM groups (53.9% vs 47.7%, p = 0.053). In contrast, IDUs had the highest rate of HCV seropositivity (94.5%), compared with heterosexuals (12.0%) and MSM (3.5%) (Table 1).

In the univariate analysis (Table 2), factors associated with anti-HAV positivity in the 2012–2016 cohort included an older age, female sex, higher PVL, residence in regions other than southern Taiwan, non-MSM, HBV infection as indicated by presence of either HBsAg, anti-HBc or both) and HCV infection (Table 2, all p<0.05). No correlation was found between the results of anti-HBs antibody and RPR with anti-HAV positivity.

**Table 2:** Factors associated with positive anti-HAV antibody in the 2012–2016 cohort.

	Anti-HAV antibody		Univariate			Multivariate		
	Negative(n = 2255)	Positive(n = 605)	OR	95% CI	p	OR	95% CI	P
Age, mean ± SD, years	31.1 ± 8.0	39.3 ± 10.8	-	-	<0.01	1.13	1.11–1.15	<0.01
Male sex, n (%)	2171 (96.3)	559 (92.4)	0.47	0.32–0.68	<0.01	0.59	0.28–1.23	0.29
CD4, mean ± SD, cells/μl	287.1 ± 188.0	277.6 ± 199.5	-	-	0.28	-	-	-
Plasma HIV RNA load, mean ± SD, log_10_ copies/ml	4.8 ± 0.8	4.7 ± 0.8	-	-	0.01	0.95	0.83–1.09	0.46
Residence in Taiwan, n (%)					<0.01			
Northern	1579 (70.0)	434 (71.7)	Referent			Referent		
Central	216 (9.6)	100 (16.5)	1.68	1.30–2.18	<0.01	0.75	0.48–1.16	0.19
Southern	460 (20.4)	71 (11.7)	0.56	0.43–0.74	<0.01	0.49	0.34–0.72	<0.01
Risk group, n (%)					<0.01			
MSM	1857 (82.3)	372 (61.5)	Referent			Referent		
Heterosexuals	104 (4.6)	62 (10.2)	2.94	2.35–3.68	<0.01	1.49	0.83–2.66	0.18
IDUs	275 (12.2)	162 (26.8)	2.98	2.13–4.15	<0.01	1.44	0.75–2.75	0.27
Others	19 (0.8)	9 (1.5)	2.37	1.06–5.27	0.03	2.861	0.76–10.85	0.12
HBV infection^a^, n (%)	427/1559 (27.4)	220/371 (59.3)	3.86	3.05–4.89	<0.01	1.44	1.08–1.93	0.01
Anti-HBs-positive, n (%)	864/1652 (52.3)	219/399 (54.9)	1.11	0.89–1.38	0.35	-	-	-
Anti-HCV-positive, n (%)	325/2249 (14.5)	186/600 (31.0)	2.66	2.16–3.28	<0.01	1.523	0.90–2.59	0.12
RPR-positive, n (%)	404/1622 (24.9)	87/391 (22.3)	0.86	0.66–1.12	0.27	-	-	-

**Abbreviations:** 95% CI, 95% confidence interval; anti-HAV, anti-hepatitis A virus; anti-HBs, anti-hepatitis B surface; anti-HBc, anti-hepatitis B core; anti-HCV, anti-hepatitis C virus; HBsAg, hepatitis B surface antigen; IDUs, injecting drug users; MSM, men who have sex with men; OR, odds ratio; RPR, rapid plasma reagin; SD, standard deviation.

^a^HBV infection indicates presence of either HBsAg, anti-HBc or both.

In the multivariate analysis (Table 2), an older age (adjusted odds ratio [AOR], per 1-year increase, 1.13; 95% confidence interval [95% CI], 1.11–1.15) and HBV infection (AOR 1.40; 95% CI, 1.03–1.90) were independently associated with HAV seropositivity. On the other hand, residents in southern Taiwan were less likely to have positive anti-HAV antibody than those in northern Taiwan (AOR 0.49; 95% CI, 0.34–0.72). To explore the association between HAV seropositivity and HBV infection, we performed a subgroup analysis by dividing our cohort into two groups including the sexually-transmitted group (heterosexuals and MSMs) (S1 Table) and the percutaneous exposure (IDUs) group (S2 Table). In the multivariate analysis, the statistically significant association between HAV and HBV infection was still noted in the sexually-transmitted group (AOR 1.38; 95% CI, 1.06–1.78), but not in the percutaneous exposure group (AOR 1.43; 95% CI, 0.62–3.30).

**Fig 1:**
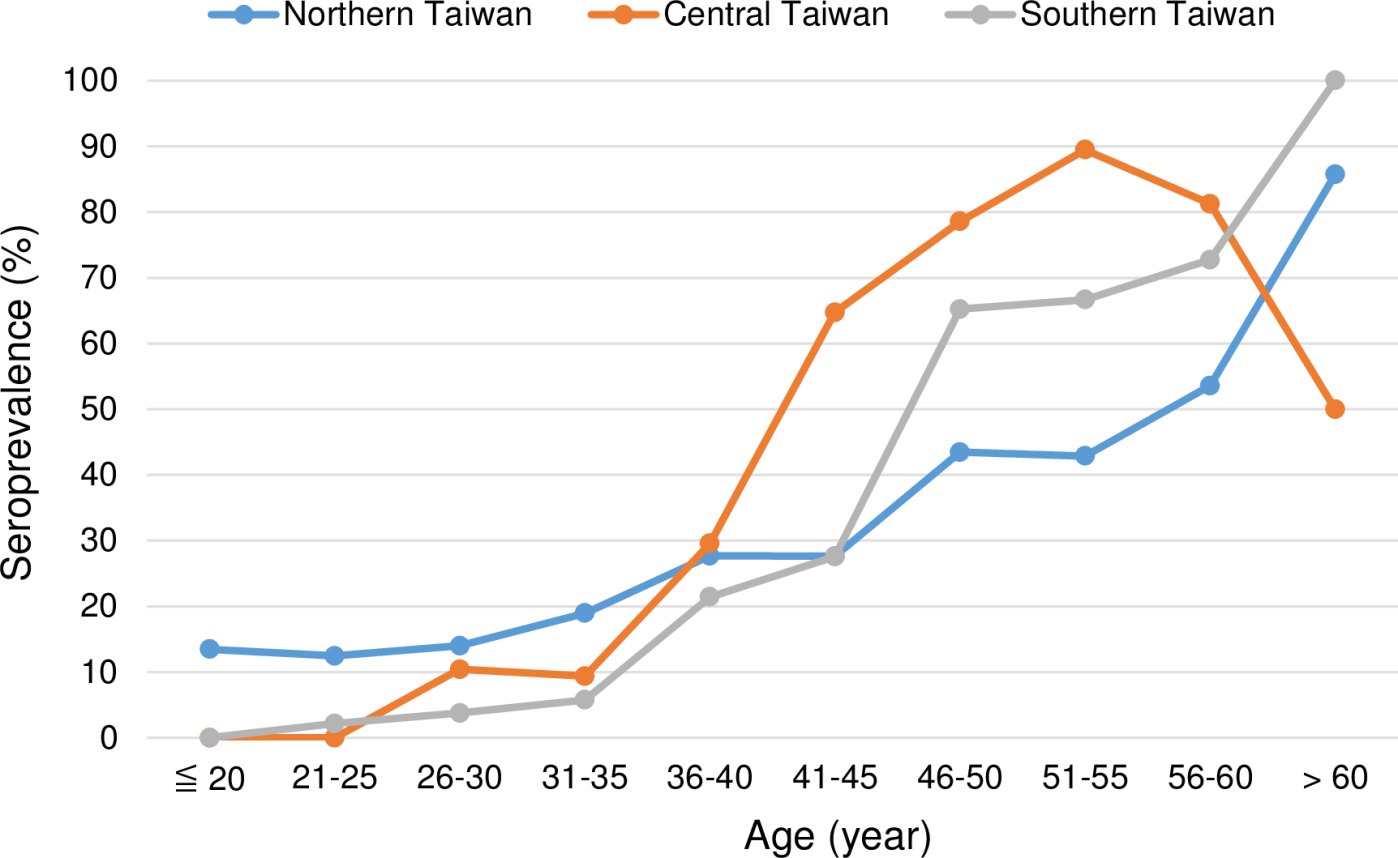
Comparisons of hepatitis A virus seroprevalence according to different regions in Taiwan.

We further examined the impact of geographic region and risk group for HIV transmission on the HAV seroprevalence in different age groups. We found that patients in central Taiwan after the age of 40–45 years and those in northern Taiwan before the age of 30–35 years had higher HAV seroprevalence than the other two regions (Fig 1). HAV seroprevalence among IDUs in central and southern Taiwan was 48.7% and 52.6%, respectively, which was significantly higher than that of northern Taiwan (29.7%). Heterosexuals in central Taiwan had higher HAV seroprevalence (55.2%) than those in northern Taiwan (34.7%) and southern Taiwan (32.3%). Conversely, MSM in northern Taiwan (19.6%) had the highest HAV seroprevalence followed by those in central Taiwan (12.3%) and those in southern Taiwan (7.2%). The higher HAV seroprevalence in the young cohort in northern Taiwan was attributed to higher HAV seroprevalence among MSM (S1 Fig). In contrast, the higher HAV seroprevalence in central Taiwan was related to higher HAV seropositivity among the older IDUs and heterosexuals (S2 and S3 Figs).

In order to investigate the evolution of HAV seroprevalence, we compared the current 2012–2016 cohort to the 2004–2007 cohort [9]. HIV-positive patients in the 2012–2016 cohort were significantly younger than those in the 2004–2007 cohort (mean age, 32.9 vs. 40.7 years, p<0.05). The proportion of patients aged less than 25 years increased from 3.3% (52/1580) in the 2004–2007 cohort to 20.5% (587/2860) in the 2012–2016 cohort. Most (95.4%, 560/587) of the young patients in the 2012–2016 cohort were MSM (Table 1). In contrast, the proportion of IDUs declined from 36.5% (577/1580) in the 2004–2007 cohort to 15.3% (437/2860) in the 2012–2016 cohort.

**Fig 2:**
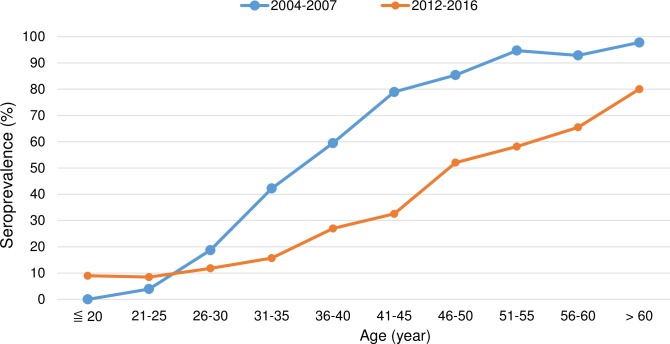
Comparisons of hepatitis A virus seroprevalence according to age-specific groups between the 2004–2007 cohort and the 2012–2016 cohort.

**Fig 3:**
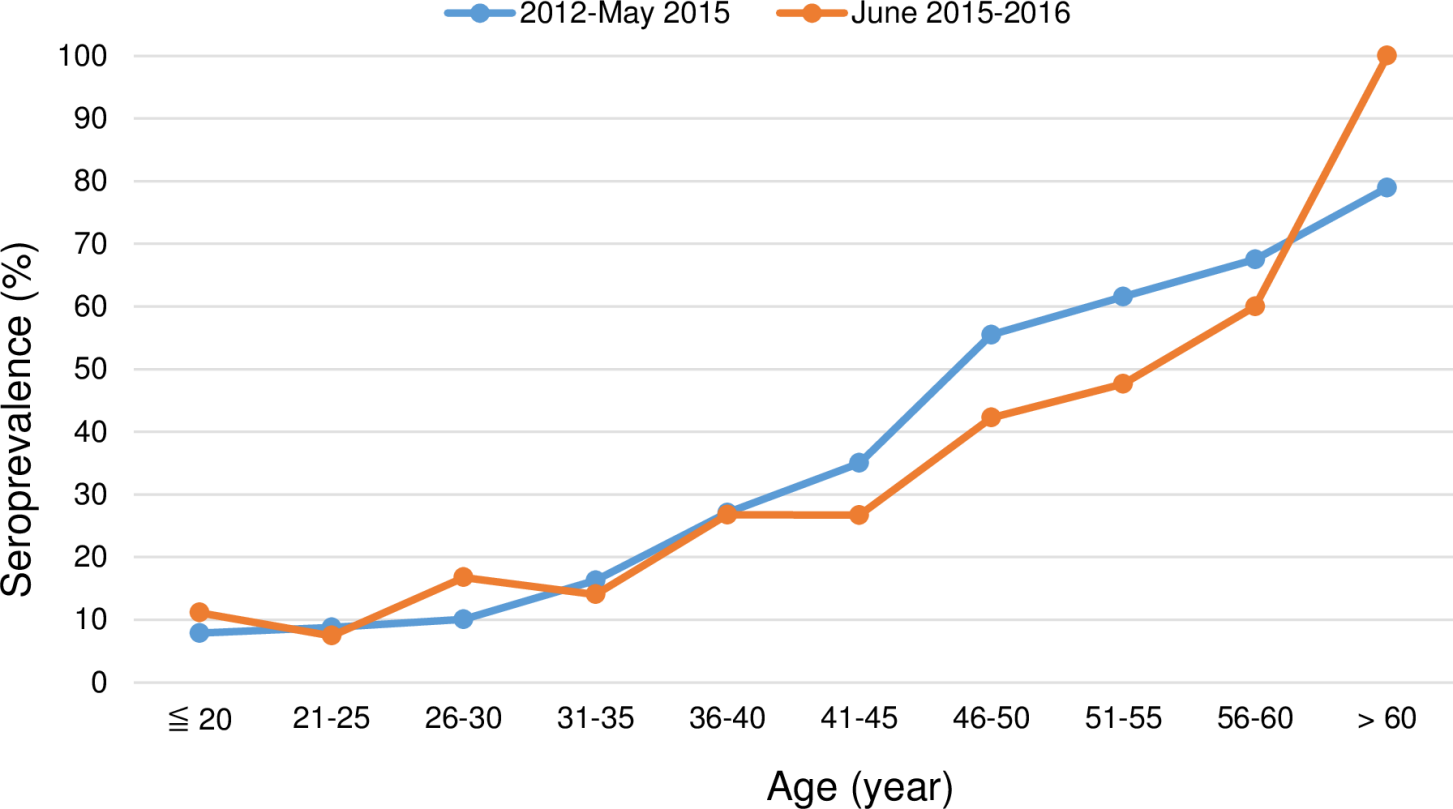
Comparisons of hepatitis A virus seroprevalence according to age-specific groups in the 2012–2016 cohort during the non-epidemic (2012—May 2015) and epidemic (June 2015–2016) period.

Although the HAV seroprevalence increased with age in both cohorts, the overall HAV seroprevalence of the 2012–2016 cohort (21.5%) was significantly lower than that of the 2004–2007 cohort (60.9%, p<0.05). Two diverse trends, however, were observed for the evolution of age-specific seroprevalence. Patients aged less than 25 years had a higher HAV seroprevalence in the 2012–2016 cohort than those in the 2004–2007 cohort; in the rest of specific age groups, a parallel decline of HAV seroprevalence was observed from the 2004–2007 cohort to the 2012–2016 cohort (all comparisons between the age-matched groups, p<0.001, Fig 2). For subgroup analysis of patients in different risk groups for HIV transmission, two different trends of HAV seroprevalence were also found in MSM and heterosexuals (S4 and S5 Figs). There were only 2 HIV-positive IDUs aged less than 25 years in the 2012–2016 cohort and the number was too small for comparison (S6 Fig). When the 2012–2016 cohort was divided into two groups including those before or after the recent outbreak of acute hepatitis A in mid-2015, a higher HAV seroprevalence was found among the individuals aged 30 years or younger who were included after June 2015 (Fig 3). Different from the findings in the 2004–2007 cohort [9], young MSM was the most at-risk population for HAV infection in the recent outbreak in Taiwan.

Comparing the HAV seroprevalence by year of birth may provide a more precise description of the cohort effect (Table 3). Persons born before 1980 who were included in the 2012–2016 cohort had a lower HAV seroprevalence than those in the 2004–2007 cohort. However, the HAV seroprevalence increased in patients born between 1980 and 1988 from 6.9% (11/159) in the 2004–2007 cohort to 13.7% (141/1027) in the 2012–2016 cohort. Moreover, the proportion of HIV-positive patients born after 1988 had significantly increased and most of the young HIV-positive patients were MSM (784/821, 95.5%) and their HAV seroprevalence was 9.9% (81/821) (Table 3).

**Table 3:** Comparisons of hepatitis A seroprevalence by age and birth year between the 2004–2007 cohort and the 2012–2016 cohort.

Study cohort	Sun et al.[9] (2004–2007 cohort)			Current study (2012–2016 cohort)		
Overall						
Age, years	Year of birth	Case/total	Rate, %	Year of birth	Case/total	Rate, %
18–20	After 1988	0/1	0	After 1996	6/35	17.1
20–28	1980–1988	11/159	6.9	1988–1996	75/786	9.5
28–36	1972–1980	182/460	39.6	1980–1988	141/1027	13.7
36–44	1964–1972	334/483	69.2	1972–1980	153/580	26.4
44–52	1956–1964	240/272	88.2	1964–1972	133/279	47.7
52–60	1948–1956	108/116	93.1	1956–1964	73/123	59.3
60–68	1940–1948	53/54	98.1	1948–1956	18/23	78.3
>68	Before 1940	34/35	97.1	Before 1948	6/7	85.7
Men who have sex with men						
18–20 years	After 1988	0/0	-	After 1996	6/31	19.4
20–28	1980–1988	2/46	4.3	1988–1996	71/753	9.4
28–36	1972–1980	41/186	22.0	1980–1988	114/898	12.7
36–44	1964–1972	121/217	55.8	1972–1980	82/357	23.0
44–52	1956–1964	85/106	80.2	1964–1972	71/149	47.7
52–60	1948–1956	28/30	93.3	1956–1964	21/33	63.6
60–68	1940–1948	8/8	100	1948–1956	6/7	85.7
>68	Before 1940	4/4	100	Before 1948	1/1	100

## Discussion

The present study demonstrated the evolution of HAV seroprevalence due to cohort effect related to the changes of HIV epidemiology in Taiwan. There were two diverse trends of HAV seroprevalence between specific age groups of HIV-positive patients in Taiwan. We observed a parallel decline of HAV seropositivity in each age-matched group older than 25 years between the two cohorts with an interval of 8 years (Fig 2). However, in the young cohort (less than 25 years) mainly consisting of MSM, HAV seroprevalence increased than before. The findings may demonstrate the impact of the changing HIV epidemiology on HAV seroepidemiology in Taiwan in recent years [23, 24], when the average age of individuals acquiring HIV has been decreasing and MSM account for the majority of the patients newly diagnosed with HIV infection.

Taiwan used to be endemic for HAV infection before 1980s [30]. HAV seroprevalence was 81.3% in the age group 20 to 24 years in 1979 in Taipei area and nearly 100% among subjects in southern Taiwan in 1981 [19–21]. With the improvement of sanitation, people born after 1982 had significant declines of HAV seroprevalence [30]; only 0.96% (2/209) among individuals born between 1984 and 1985 tested positive for anti-HAV antibodies in the adolescents [31]. The cohort effect was also observed in the HIV-positive patients. In this study, we found that the age of 50% HAV seropositivity among HIV-positive patients had shifted from 35–40 years to 45–50 years between the two cohorts with 8 years apart (Fig 2). The decreasing immunity against HAV has created a huge number of susceptible host to acquisition of HAV, especially in the young cohort. As a result, the surveillance data from Taiwan CDC revealed that the majority of the HIV-positive patients acquiring acute hepatitis A in the recent outbreak since June 2015 were men aged between 18 and 39 years who contracted HIV through unsafe sex [32].

Although HAV infection is a vaccine-preventable disease, cases of acute hepatitis A continue to occur worldwide because of low awareness of and adherence to HAV vaccination [27, 33]. A recent outbreak of acute hepatitis A in the Netherlands between July 2016 and February 2017 was linked to an international event to celebrate equality rights of the lesbian, gay, bisexual and transgender community called “EuroPride” that took place in Amsterdam in 2016 [17]. According to the phylogenic analysis of HAV sequencing, the EuroPride strain RIVM-HAV16-090 was 99.57% identical to the previous strain submitted by Japan in 2001 and the strain responsible for the recent outbreak of acute hepatitis A in Taiwan [34, 35]. In addition, the strain also caused several HAV outbreaks in the United Kingdom, Germany, Italy, and Spain since late 2016. The worldwide outbreaks have two features in common: all of the affected countries were of low endemicity for HAV infection, and most of the patients in the outbreaks were young MSM [16–18]. The international travel and unprotected sexual contacts among MSM populations, including oral-anal sex or digital-anal sex, might have played an important role in the transmission of HAV.

In our 2012–2016 cohort, the association between injecting drug use and higher HAV prevalence was no longer observed. In Taiwan, with the implementation of harm reduction program since 2004–2005 [25, 36] comes with the significant decreases of IDUs acquiring HIV in recent years. The IDUs accounted for 15.3% of all HIV-positive patients in the 2012–2016 cohort, which was significantly lower than that (36.5%) in the 2004–2007 cohort [9]; moreover, the HAV seroprevalence among IDUs also decreased from 62.0% to 37.1% despite the average age increased from 35.7 years to 41.8 years. We postulate that the harm reduction program that included expanded access to counseling, screening, clean needles and syringes, and methadone maintenance treatment [25] might have not only changed the HIV epidemiology but decreased HAV transmission among IDUs in Taiwan.

An association between HBV and HAV infection was noted among MSM and heterosexuals, but not IDUs in our multivariate analysis. In previous studies on MSM, several factors such as the number of sexual partners, group sex, oral-anal and digital-rectal intercourse were associated with both HAV and HBV infection [6, 37]. The findings in our subgroup analysis also suggested the intimate contact such as sex exposure may increase both of the risks of HAV and HBV transmission. While the factors such as poor personal hygiene, oral ingestion of faecally-contaminated drugs and parenteral transmission have been identified to facilitate HAV transmission among IDUs [38], HBV and HCV have higher infectivity through parenteral routes than HAV. Most of our IDUs were born in the era without vaccination coverage [9, 37] and the rate of HBV infection had been high regardless of the positive result of anti-HAV antibody (50.0%) or not (46.0%). Similar to previous study, as high as 94.5% of IDUs had HCV infection in out cohort [9]. The high infection rates of HBV and HCV infection among the IDUs may preclude us from identifying the association between HAV seropositivity and HCV or HBV infection.

HAV vaccination is the most effective strategy in preventing HAV infection [39, 40]. Many countries including Israel, U.S.A., Argentina and Chile have introduced universal HAV vaccination in routine childhood immunizations and have achieved great reduction of HAV infection in the general population [41–44]. Besides, the cost-effective analysis for universal childhood HAV vaccination also demonstrated both health and economic benefits [45–47]. The previous vaccination policy in Taiwan covered only 2% of the total population, however [48]. Given the fact that a mathematical model suggested an immune threshold of 70% to prevent HAV outbreaks [49], Taiwan is at high risk of outbreaks of acute HAV infection because of increasing numbers of susceptible hosts and frequency of international travel. The adherence to recommendations of HAV vaccination was low among the HIV-positive MSM in recent surveys [27, 28], and implementation of nationwide HAV vaccination program is urgently needed to control and prevent the HAV outbreaks.

There are several limitations of our study. First, the included patients came from different areas in these two cohorts used to examine the evolution of HAV seroprevalence. The present 2012–2016 cohort included HIV-positive patients from 11 designated hospitals for HIV care around Taiwan while the previous 2004–2007 cohort included patients from only two hospitals located in northern and central Taiwan. To minimize the interference of geographic variation on the HAV seroprevalence, we performed a sensitivity analysis by comparing only patients from northern and central Taiwan. The evolution of HAV seroprevalence was still noted (S7 Fig). Second, the information on personal hygiene, living environment, socioeconomic status, sexual behaviors, and illicit drug-taking behavior was lacking in our study. Those factors may confound the findings of changing HAV prevalence. Third, we were not able to collect the history of HAV vaccination in this retrospective study. Some of our patients might have been vaccinated and presence of anti-HAV antibody could not be used to differentiate natural infection from vaccination, though a recent survey suggested that the rate of HAV vaccination was low before the recent outbreak of acute hepatitis A among MSM in Taiwan [27].

In conclusion, the HAV seroepidemiology in HIV-positive patients is changing in Taiwan. The cohort effect has created a huge number of susceptible host to HAV infection, which may have contributed to the outbreak of acute hepatitis A among young HIV-positive MSM in Taiwan in recent years. Information, education and communication to increase the HAV vaccination coverage are urgently needed among the susceptible individuals.

## Supporting information

S1 Fig Comparisons of hepatitis A virus seroprevalence among men who have sex with men (MSM) according to geographic regions in Taiwan. (TIF)

S2 Fig Comparisons of hepatitis A virus seroprevalence among injecting drug users (IDUs) according to geographic regions in Taiwan. (TIF)

S3 Fig Comparisons of hepatitis A virus seroprevalence among heterosexuals according to geographic regions in Taiwan. (TIF)

S4 Fig Comparisons of hepatitis A virus seroprevalence according to age-specific groups among men who have sex with men between the 2004–2007 cohort and the 2012–2016 cohort. (TIF)

S5 Fig Comparisons of hepatitis A virus seroprevalence according to age-specific groups among heterosexuals between the 2004–2007 cohort and the 2012–2016 cohort. (TIF)

S6 Fig Comparisons of hepatitis A virus seroprevalence according to age-specific groups among injecting drug users between the 2004–2007 cohort and the 2012–2016 cohort. (TIF)

S7 Fig Sensitivity analysis of comparing hepatitis A seroprevalence according to age-specific groups between the 2004–2007 cohort and the 2012–2016 cohort including only patients from northern and central Taiwan. (TIF)

S1 Table Factors associated with positive anti-HAV antibody among men who have sex with men (MSM) and heterosexuals. (DOCX)

S2 Table Factors associated with positive anti-HAV antibody among injecting drug users (IDUs). (DOCX)

S3 Table Comparisons of hepatitis A virus seroprevalence by age and birth year among heterosexuals in the two cohorts. (DOCX)

S4 Table Comparison of hepatitis A virus seroprevalence by age and birth year among injecting drug users (IDUs) in the two cohorts. (DOCX)

S1 Data The minimal data set of the patients in this study. (XLSX)
