# Supplementary figures and images for: Evolution of hepatitis A virus seroprevalence among HIV-positive adults in Taiwan
Source: PLoS One. 2017 Oct 16;12(10):e0186338. doi: 10.1371/journal.pone.0186338 (PMC5643057; doi:10.1371/journal.pone.0186338)

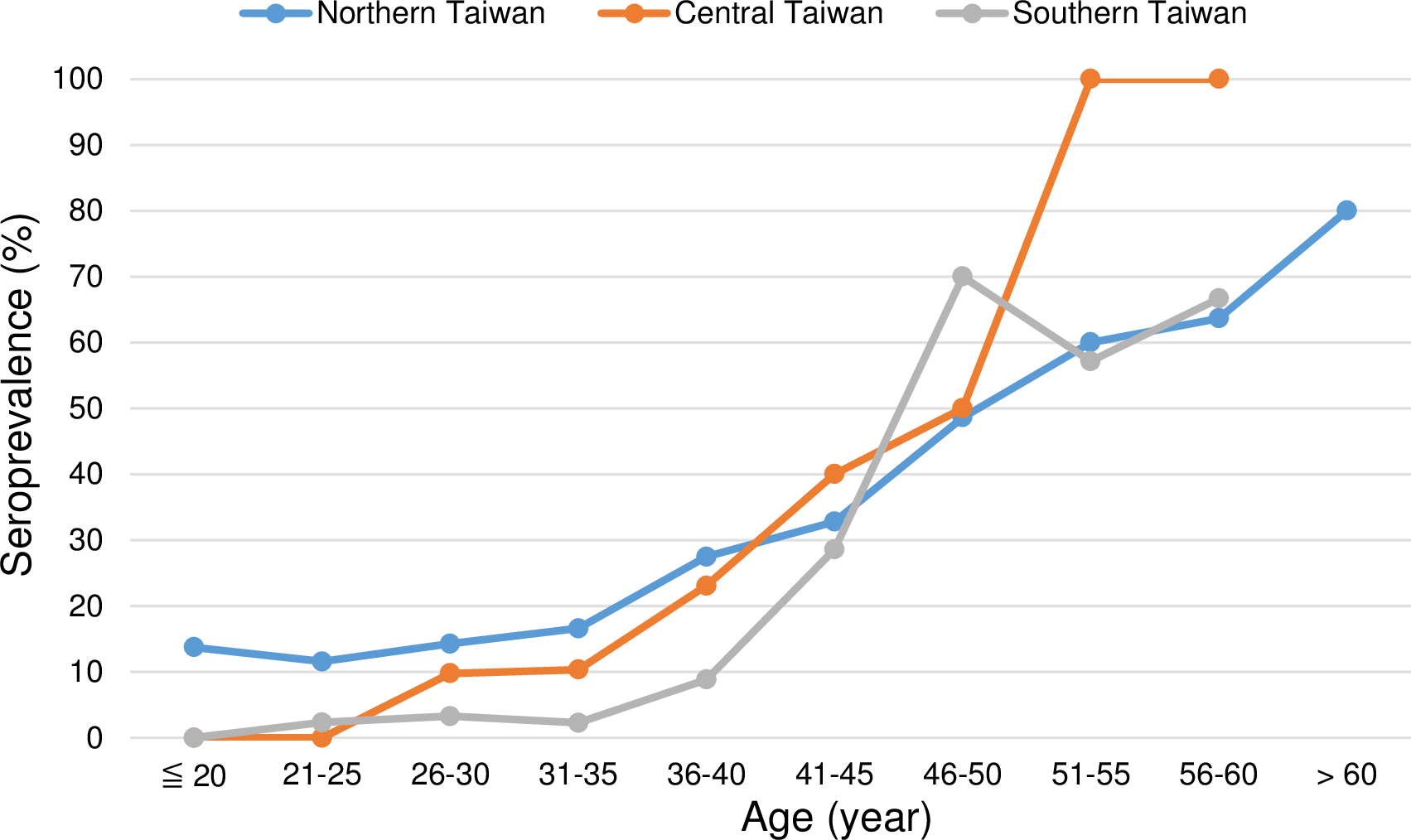

Supplement: S1 Fig — (TIF) [file pone.0186338.s001.tif]

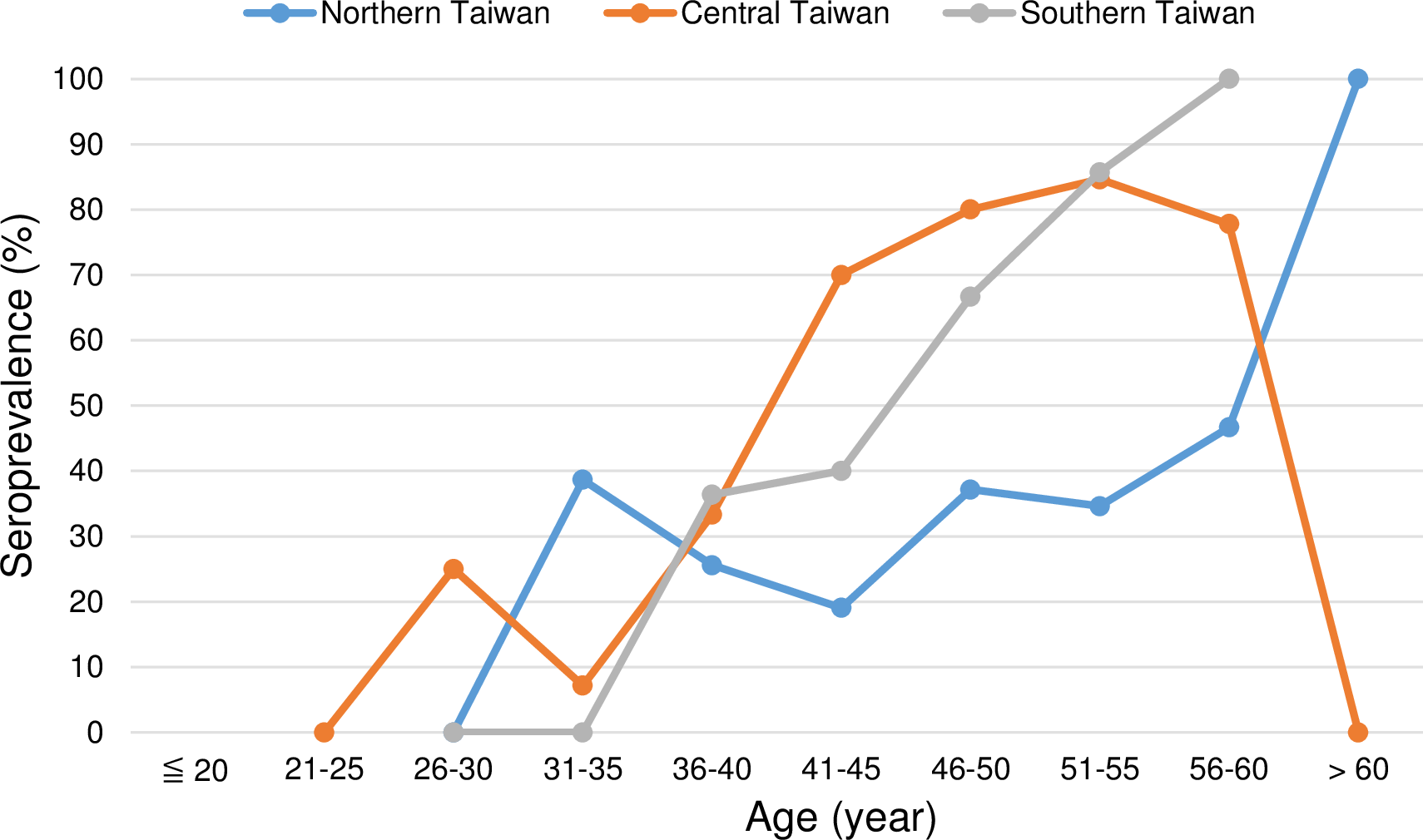

Supplement: S2 Fig — (TIF) [file pone.0186338.s002.tif]

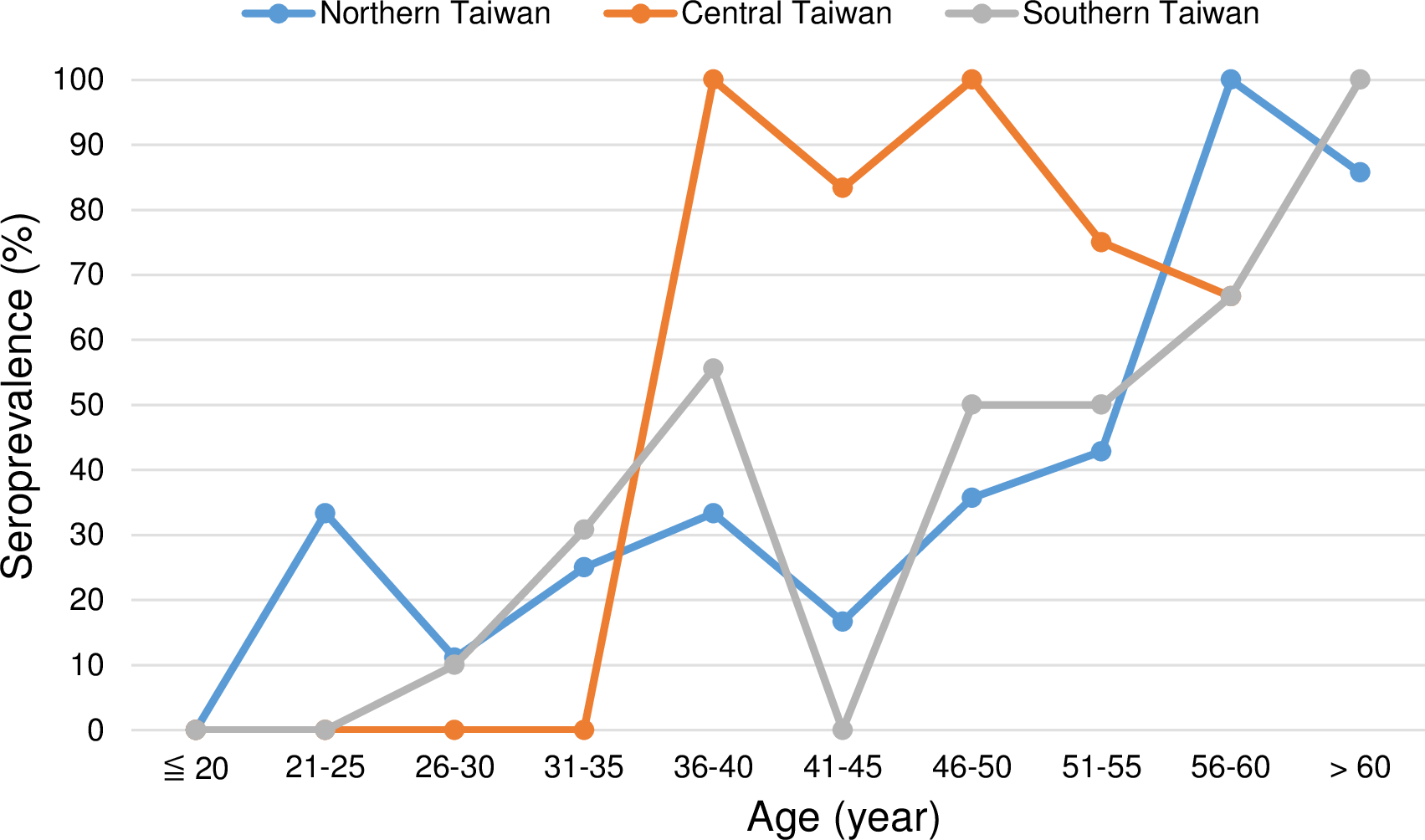

Supplement: S3 Fig — (TIF) [file pone.0186338.s003.tif]

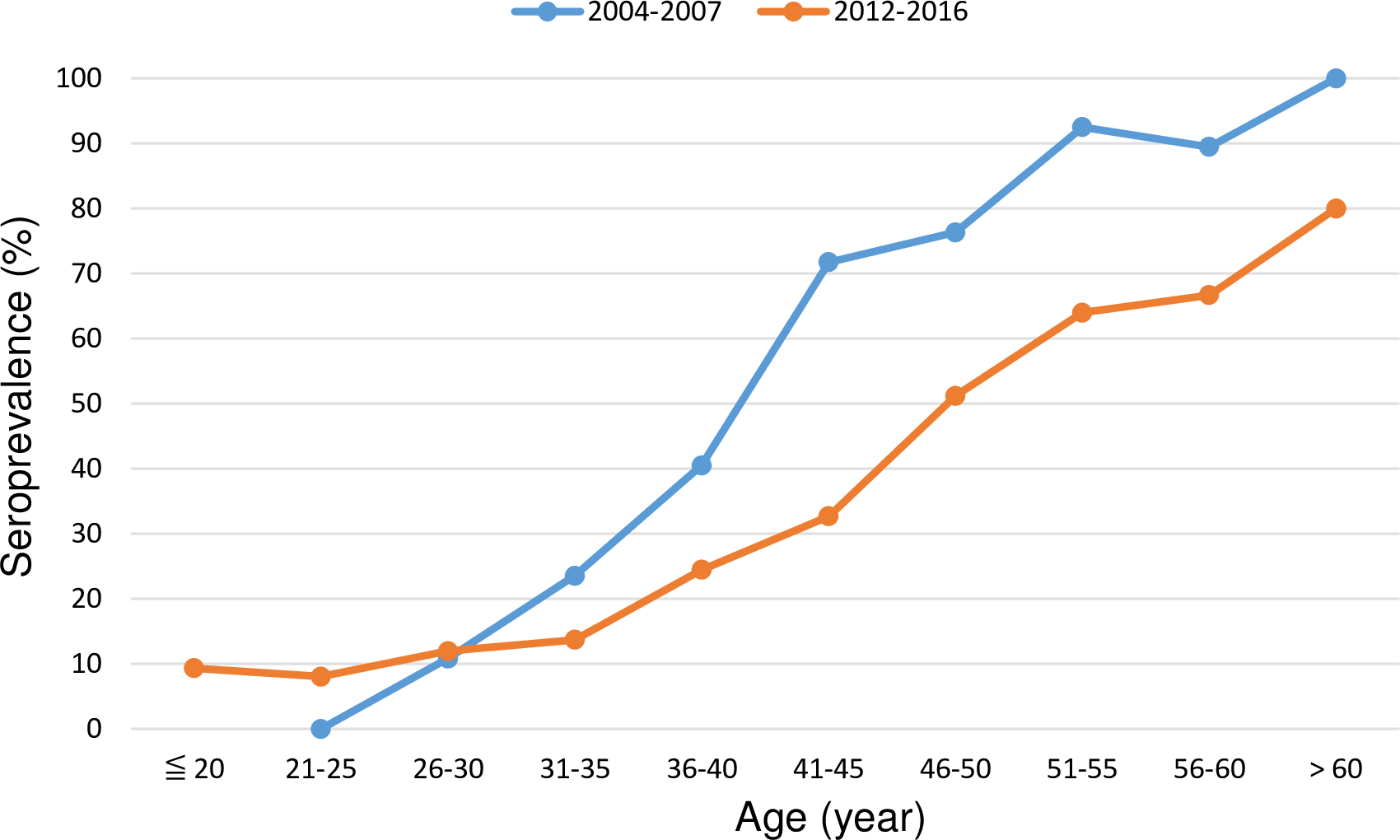

Supplement: S4 Fig — (TIF) [file pone.0186338.s004.tif]

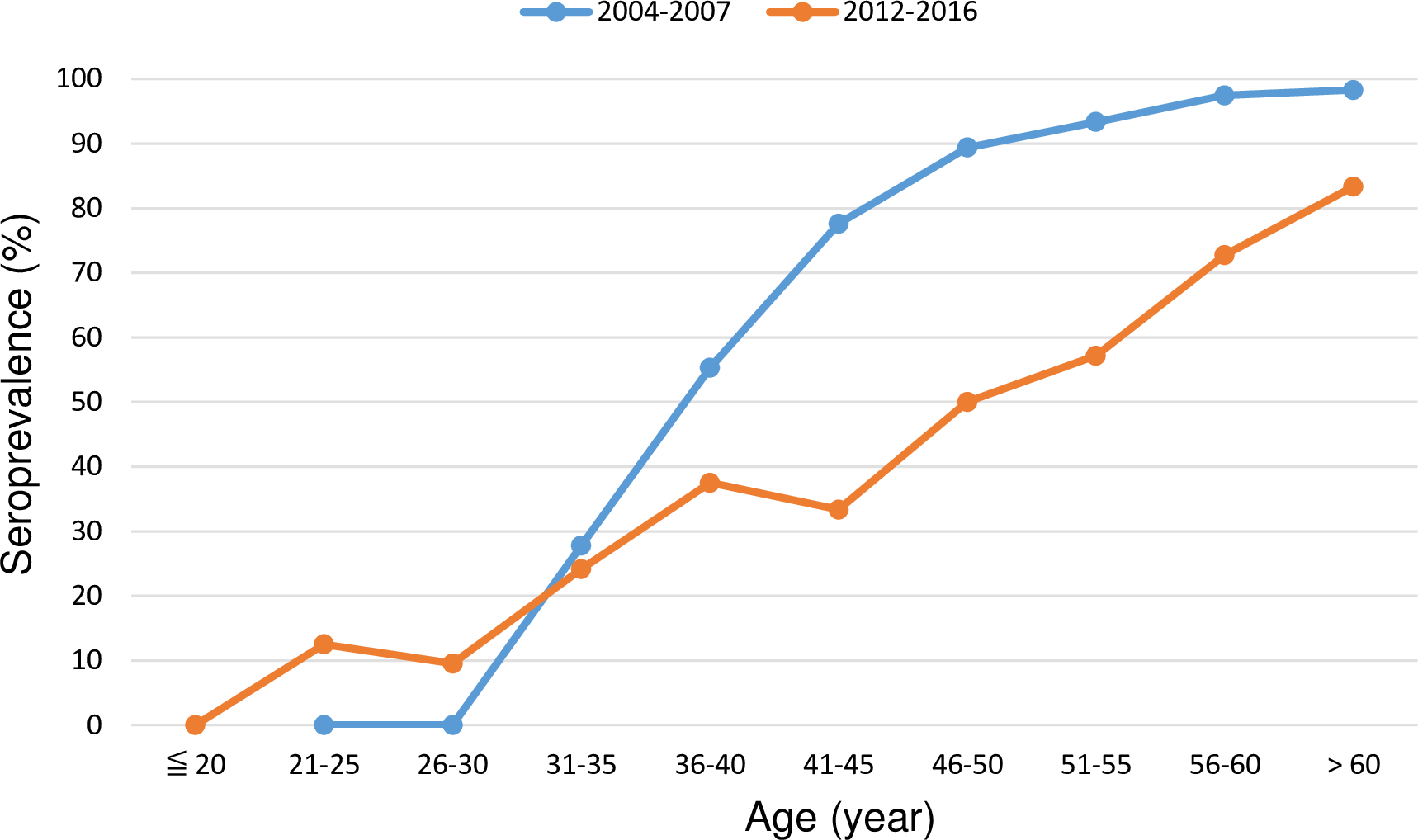

Supplement: S5 Fig — (TIF) [file pone.0186338.s005.tif]

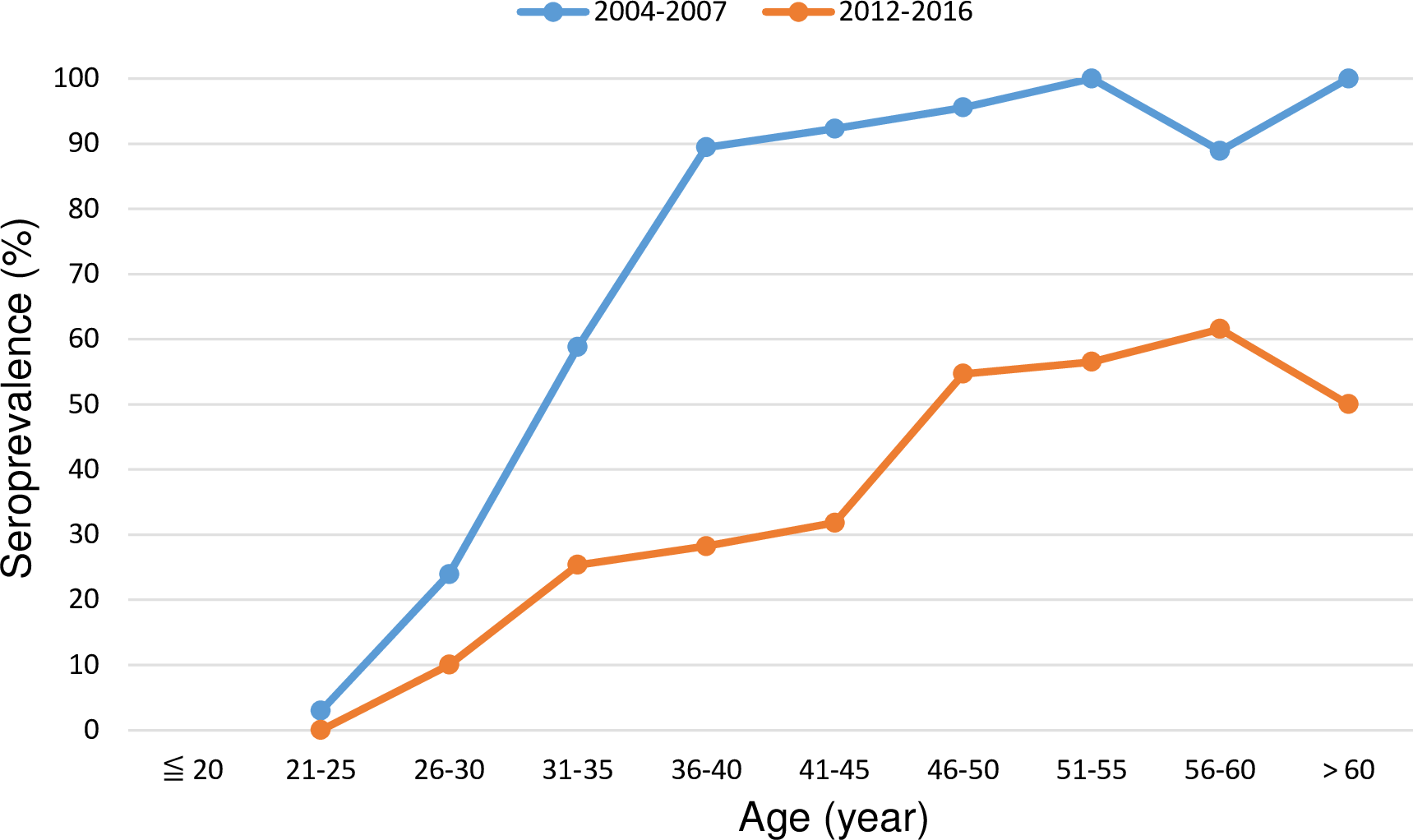

Supplement: S6 Fig — (TIF) [file pone.0186338.s006.tif]

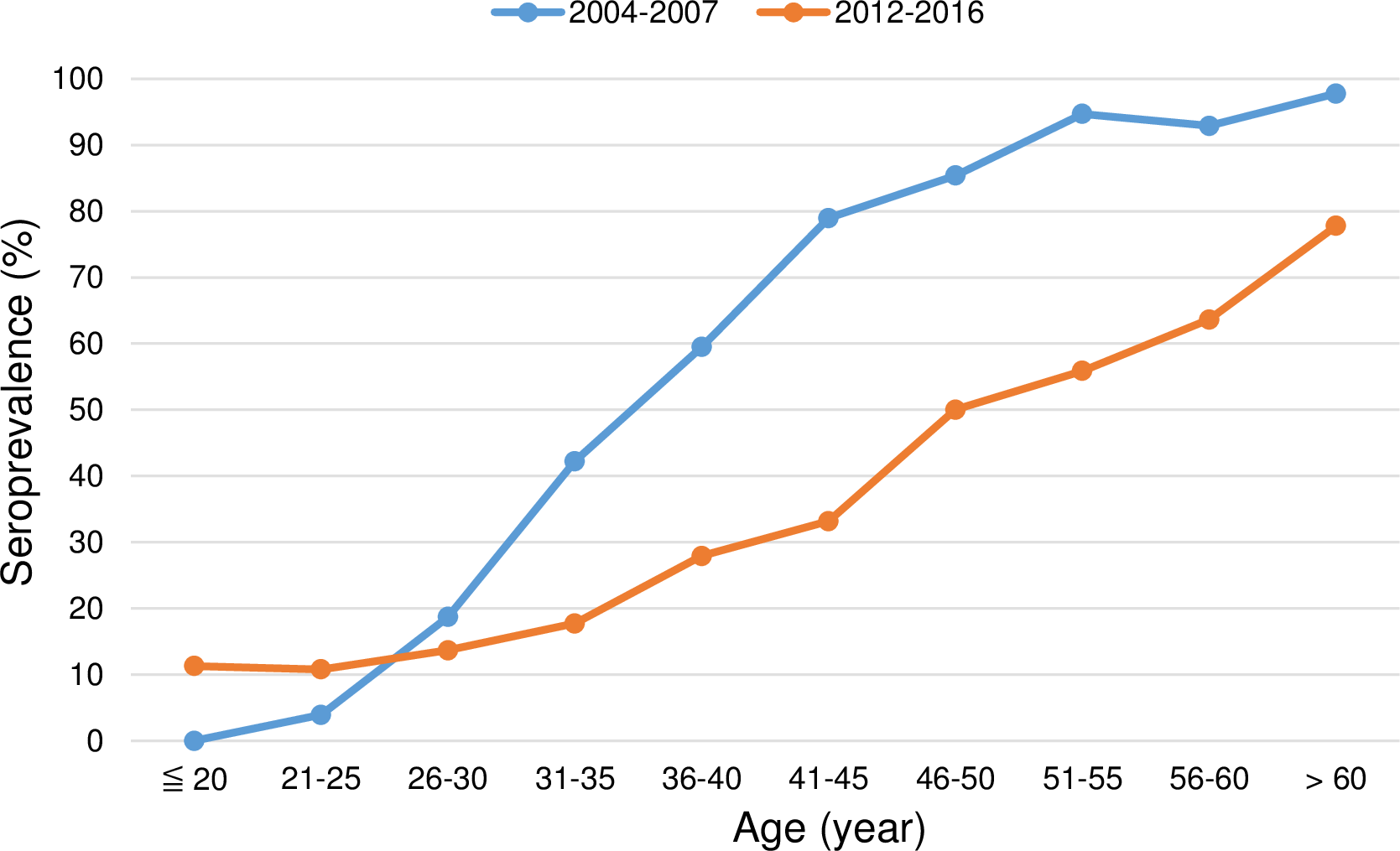

Supplement: S7 Fig — (TIF) [file pone.0186338.s007.tif]
