# Supplementary material for: Evolution of hepatitis A virus seroprevalence among HIV-positive adults in Taiwan
Source: PLoS One. 2017 Oct 16;12(10):e0186338. doi: 10.1371/journal.pone.0186338 (PMC5643057; doi:10.1371/journal.pone.0186338)
Supplement: S1 Table — (DOCX) [file pone.0186338.s008.docx]

|  | Anti-HAV antibody | |  | Univariate | | |  | Multivariate | | |
| --- | --- | --- | --- | --- | --- | --- | --- | --- | --- | --- |
|  | Negative  (n=1961) | Positive  (n=434) |  | OR | 95% CI | p |  | OR | 95% CI | P |
| Age, mean ± SD, years | 29.8 ± 7.2 | 37.4 ± 11.0 |  | - | - | <0.01 |  | 1.09 | 1.08-1.11 | <0.01 |
| Male sex, n (%) | 1930 (98.4) | 406 (93.5) |  | 0.23 | 0.14-0.39 | <0.01 |  | 0.50 | 0.23-1.08 | 0.08 |
| CD4, mean± SD, cells/μl | 282.5 ± 191.1 | 282.7 ± 212.4 |  | - | - | 0.99 |  | - | - | - |
| Plasma HIV RNA load, mean ± SD, log_10_ copies/ml | 4.84 ± 0.74 | 4.75 ± 0.77 |  | - | - | 0.02 |  |  |  |  |
| Residence, n (%) |  |  |  |  |  | <0.01 |  |  |  |  |
| Northern Taiwan | 1374 (70.1) | 348 (80.2) |  | Referent |  |  |  | Referent |  |  |
| Central Taiwan | 148 (7.5) | 35 (8.1) |  | 0.93 | 0.63-1.38 | 0.73 |  | 0.68 | 0.43-1.08 | 0.10 |
| Southern Taiwan | 439 (22.4) | 51 (11.8) |  | 0.46 | 0.34-0.63 | <0.01 |  | 0.45 | 0.31-0.64 | 0.01 |
| HBV infection^a^, n (%) | 418/1957 (21.4) | 171/432 (39.6) |  | 2.41 | 1.93-3.01 | <0.01 |  | 1.38 | 1.06-1.78 | 0.02 |
| Anti-HBs-positive, n (%) | 821/1554 (52.8) | 170/301 (56.5) |  | 1.16 | 0.90-1.49 | 0.25 |  | - | - | - |
| Anti-HCV-positive, n (%) | 66/1956 (3.4) | 32/429 (7.5) |  | 2.31 | 1.49-3.57 | <0.01 |  | 1.72 | 1.05-2.80 | 0.03 |
| RPR-positive, n (%) | 376/1523 (24.7) | 70/295 (23.7) |  | 0.95 | 0.71-1.27 | 0.70 |  | - | - | - |

**S1 table. Factors associated with positive anti-HAV antibody among men who have sex with men (MSM) and heterosexuals**

**Abbreviations:** 95% CI, 95% confidence interval; anti-HAV, anti-hepatitis A virus; anti-HBs, anti-hepatitis B surface; anti-HBc, anti-hepatitis B core; anti-HCV, anti-hepatitis C virus; RPR, rapid plasma reagin; SD, standard deviation

^a^HBV infection indicates presence of either HBsAg, anti-HBc antibody or both
