# Supplementary material for: Evolution of hepatitis A virus seroprevalence among HIV-positive adults in Taiwan
Source: PLoS One. 2017 Oct 16;12(10):e0186338. doi: 10.1371/journal.pone.0186338 (PMC5643057; doi:10.1371/journal.pone.0186338)
Supplement: S2 Table — (DOCX) [file pone.0186338.s009.docx]

**S2 table. Factors associated with positive anti-HAV antibody among injecting drug users**

|  | Anti-HAV antibody | |  | Univariate | | |  | Multivariate | | |
| --- | --- | --- | --- | --- | --- | --- | --- | --- | --- | --- |
|  | Negative  (n=275) | Positive  (n=162) |  | OR | 95% CI | p |  | OR | 95% CI | P |
| Age, mean ± SD, years | 40.3 ± 7.1 | 44.3 ± 8.1 |  | - | - | <0.01 |  | 1.16 | 1.10-1.23 | <0.01 |
| Male sex, n (%) | 223 (81.1) | 145 (89.5) |  | 1.99 | 1.11-3.57 | 0.02 |  | 1.230 | 0.28-5.36 | 0.78 |
| CD4, mean± SD, cells/μl | 326.4 ± 159.6 | 266.1 ± 155.8 |  | - | - | <0.01 |  | 0.999 | 0.995-1.002 | 0.48 |
| Plasma HIV RNA load, mean ± SD, log_10_ copies/ml | 4.2 ± 0.8 | 4.5 ± 0.9 |  | - | - | 0.01 |  | 1.25 | 0.82-1.91 | 0.29 |
| Residence, n (%) |  |  |  |  |  | <0.01 |  |  |  |  |
| Northern Taiwan | 194 (70.6) | 82 (50.7) |  | Referent |  |  |  | Referent |  |  |
| Central Taiwan | 63 (22.9) | 60 (37.0) |  | 2.25 | 1.45-3.49 | <0.01 |  | 0.83 | 0.24-2.82 | 0.76 |
| Southern Taiwan | 18 (6.5) | 20 (12.3) |  | 2.63 | 1.32-5.23 | 0.05 |  | 0.57 | 0.14-2.37 | 0.43 |
| HBV infection^a^, n (%) | 91 (33.1) | 79 (48.8) |  | 1.93 | 1.29-2.86 | 0.01 |  | 1.43 | 0.62-3.30 | 0.40 |
| Anti-HBs-positive, n (%) | 40/87 (46.0) | 46/92 (50.0) |  | 1.18 | 0.65-2.11 | 0.59 |  | - | - | - |
| Anti-HCV-positive, n (%) | 258/274 (94.2) | 154/162 (95.1) |  | 1.19 | 0.50-2.86 | 0.69 |  | - | - | - |
| RPR-positive, n (%) | 25/88 (28.4) | 16/90 (17.8) |  | 0.55 | 0.27-1.11 | 0.09 |  | 0.360 | 0.14-0.90 | 0.03 |

**Abbreviations:** 95% CI, 95% confidence interval; anti-HAV, anti-hepatitis A virus; anti-HBs, anti-hepatitis B surface; anti-HBc, anti-hepatitis B core; anti-HCV, anti-hepatitis C virus; RPR, rapid plasma regain; SD, standard deviation

^a^HBV infection = patients with positive result of either HBsAg or anti-HBc antibody or both
