# Supplementary material for: Evolution of hepatitis A virus seroprevalence among HIV-positive adults in Taiwan
Source: PLoS One. 2017 Oct 16;12(10):e0186338. doi: 10.1371/journal.pone.0186338 (PMC5643057; doi:10.1371/journal.pone.0186338)
Supplement: S3 Table — (DOCX) [file pone.0186338.s010.docx]

**S3 table. Comparisons of hepatitis A virus seroprevalence by age and birth year among heterosexuals in the two cohorts**

| Study cohort | Sun et al.,  (2004-2007) | | | Current study  (2012-2016) | | |
| --- | --- | --- | --- | --- | --- | --- |
| Age (years) | Year of birth | Case/total | Rate, % | Year of birth | Case/total | Rate, % |
| ≦20 | After 1988 | 0/0 | - | After 1996 | 0/3 | 0 |
| 20-28 | 1980-1988 | 0/5 | 0.0 | 1988-1996 | 2/24 | 8.3 |
| 28-36 | 1972-1980 | 7/27 | 25.9 | 1980-1988 | 8/42 | 19.0 |
| 36-44 | 1964-1972 | 58/86 | 67.4 | 1972-1980 | 12/32 | 37.5 |
| 44-52 | 1956-1964 | 67/73 | 91.8 | 1964-1972 | 14/28 | 50.0 |
| 52-60 | 1948-1956 | 52/55 | 94.5 | 1956-1964 | 13/21 | 61.9 |
| 60-68 | 1940-1948 | 32/33 | 97.0 | 1948-1956 | 9/11 | 81.9 |
| >68 | Before 1940 | 25/25 | 100 | Before 1948 | 4/5 | 80 |
