# Supplementary material for: Evolution of hepatitis A virus seroprevalence among HIV-positive adults in Taiwan
Source: PLoS One. 2017 Oct 16;12(10):e0186338. doi: 10.1371/journal.pone.0186338 (PMC5643057; doi:10.1371/journal.pone.0186338)
Supplement: S4 Table — (DOCX) [file pone.0186338.s011.docx]

**S4 Table. Comparison of hepatitis A virus seroprevalence by age and birth year among injecting drug users in the two cohorts**

| Study cohort | Sun et al.,  (2004-2007) | | | Current study  (2012-2016) | | |
| --- | --- | --- | --- | --- | --- | --- |
| Age (years) | Year of birth | Case/total | Rate, % | Year of birth | Case/total | Rate, % |
| ≦20 | After 1988 | 0/0 | - | After 1996 | 0/1 | 0 |
| 20-28 | 1980-1988 | 8/105 | 4.3 | 1988-1996 | 1/2 | 50 |
| 28-36 | 1972-1980 | 130/233 | 55.8 | 1980-1988 | 18/79 | 22.8 |
| 36-44 | 1964-1972 | 139/155 | 89.7 | 1972-1980 | 56/185 | 30.3 |
| 44-52 | 1956-1964 | 63/65 | 96.9 | 1964-1972 | 47/100 | 47 |
| 52-60 | 1948-1956 | 17/18 | 94.4 | 1956-1964 | 37/65 | 56.9 |
| 60-68 | 1940-1948 | 1/1 | 100 | 1948-1956 | 2/4 | 50 |
| >68 | Before 1940 | 0/0 | - | Before 1948 | 1/1 | 100 |
